# Concept and application of the probability of pharmacological success (PoPS) as a decision tool in drug development: a position paper

**DOI:** 10.1186/s12967-022-03849-y

**Published:** 2023-01-11

**Authors:** Chao Chen, Xuan Zhou, Silvia Maria Lavezzi, Usman Arshad, Raman Sharma

**Affiliations:** 1 Clinical Pharmacology Modelling and Simulation, GSK, London, UK; 2 Clinical Pharmacology, Modelling and Simulation, Parexel International, Dublin, Ireland

**Keywords:** Drug development, Translational pharmacology, Probability of pharmacological success

## Abstract

**Background:**

In drug development, few molecules from a large pool of early candidates become successful medicines after demonstrating a favourable benefit-risk ratio. Many decisions are made along the way to continue or stop the development of a molecule. The probability of pharmacological success, or PoPS, is a tool for informing early-stage decisions based on benefit and risk data available at the time.

**Results:**

The PoPS is the probability that most patients can achieve adequate pharmacology for the intended indication while minimising the number of subjects exposed to safety risk. This probability is usually a function of dose; hence its computation typically requires exposure–response models for pharmacology and safety. The levels of adequate pharmacology and acceptable risk must be specified. The uncertainties in these levels, in the exposure–response relationships, and in relevant translation all need to be identified. Several examples of different indications are used to illustrate how this approach can facilitate molecule progression decisions for preclinical and early clinical development. The examples show that PoPS assessment is an effective mechanism for integrating multi-source data, identifying knowledge gaps, and forcing transparency of assumptions. With its application, translational modelling becomes more meaningful and dose prediction more rigorous. Its successful implementation calls for early planning, sound understanding of the disease-drug system, and cross-discipline collaboration. Furthermore, the PoPS evolves as relevant knowledge grows.

**Conclusion:**

The PoPS is a powerful evidence-based framework to formally capture multiple uncertainties into a single probability term for assessing benefit-risk ratio. In GSK, it is now expected for governance review at all early-phase decision gates.

## Background

Drug development is a process of attrition and of cost escalation [1–4]. At the portfolio level, only a small proportion of drug candidates reach later stage of development. At the asset level, the cost of development increases drastically towards later stages. The greater attrition in the early phases and the cost associated with the late-phase development together make the soundness of decisions at early stage particularly important.

A decision whether to continue investing resources in an asset must be made at each development phase. Such decisions are always made with uncertainty in the potential that the asset under investigation can eventually become an effective and safe medicine. This uncertainty is naturally greater for decisions related to the earlier stages of asset development, when there is very little, if any, direct evidence of efficacy and safety in the intent-to-treat patients. Therefore, there is a need to assess the overall pharmacological strength of the asset considering the collective uncertainties.

In this position paper, we aim to describe how the probability of pharmacological success (PoPS) can be a benefit-risk assessment tool to aid the progression decisions for early-phase compounds. The objective is to explain the key principles for its implementation and to illustrate how these principles can be applied, using diverse examples in different indications and development phases.

## General framework of PoPS

### The concept

The PoPS was first proposed as a framework to inform early-stage decisions [5]. An earlier report showed the idea in its nascent form [6]. An application to preclinical candidate selection (CS) and commit to first-in-human (FiH) was later published [7].

It is the probability that adequate pharmacology is achieved in most patients while minimizing the number of patients exposed to safety risk, given the multiple uncertainties. It is specified in the context of a drug-disease system, where the uncertainties can be broadly categorised as those related to the drug (such as its potency), the disease (such as the required level of intervention), and their interface (such as the translation of exposure–response relationship of the drug to the patient population). The specific definition depends on its situational application, as illustrated in the four case studies.

### Mathematical representation

For a given dosage regimen, the pharmacokinetic (PK) and/or pharmacodynamic (PD) endpoints used for computing PoPS can be described as:

1 $${k}_{j}=f\left(\varvec{\vartheta },{{\varvec{z}}}_{{\varvec{j}}}, {{\varvec{\eta}}}_{{\varvec{j}}}\right),\quad j=1,...,N, \quad {\varvec{\eta}}\sim N(\varvec{0},{\varvec{\Omega}})$$

In Eq. 1, ${k}_{j}$ represents the PK or PD endpoint for subject *j* (e.g., AUC or target inhibition), which is expressed as a function $f$ of fixed effects $\varvec{\vartheta }$﻿ (e.g., typical value for clearance or drug potency), individual covariates ${{\varvec{z}}}_{{\varvec{j}}}$ (e.g., weight or disease status), and inter-subject-variability ${{\varvec{\eta}}}_{{\varvec{j}}}$.

Success criteria for safety and/or efficacy can be then expressed as:

2 $${k}_{j}>\left(or \ge or <or \le \right) K \, for \, n\% \, of \, subjects$$

where $K$ is the threshold level for the PK and/or PD endpoint; and $n$ is the percentage of subjects required to meet the threshold.

Uncertainties in parameter estimation and translation (Eq. 1) can be described respectively as:

3 $$\varvec{\vartheta } \sim {d}_{1}\left({{\varvec{\lambda}}}_{1}\right), \quad {\varvec{\Omega}}\sim {d}_{2}({{\varvec{\Lambda}}}_{2})$$

Uncertainty in the success criteria (see Eq. 2) can be described as:

4 $$K \sim {d}_{3}\left({\lambda }_{3}\right), \quad n \sim {d}_{4}({\lambda }_{4})$$

In Eq. 3 and Eq. 4, ${d}_{1}$, ${d}_{2}$, ${d}_{3}$, and ${d}_{4}$ are probability distributions, respectively with parameter sets ${{\varvec{\lambda}}}_{1}$, ${{\varvec{\Lambda}}}_{2}$, ${{\varvec{\lambda}}}_{3}$, and ${{\varvec{\lambda}}}_{4}$.

PoPS calculation is then performed as follows:

- Fixed and/or random effect parameters are simulated for $M$ (e.g. 500) times as per Eq. 3 (${\varvec{\vartheta }}_{1}, \dots , {\varvec{\vartheta }}_{{\varvec{M}}}$ and ${{\varvec{\Omega}}}_{1}, \dots , {{\varvec{\Omega}}}_{{\varvec{M}}}$).
- For each set of parameters (${\varvec{\vartheta }}_{{\varvec{i}}} ,{{\varvec{\Omega}}}_{{\varvec{i}}})$,$i=1,\dots ,M$, a virtual population of $N$ (e.g. 1000) subjects is simulated and PK and/or PD endpoints are obtained as per Eq. 1 (${\left({k}_{j}\right)}_{1},\dots {\left({k}_{j}\right)}_{M}$,$j=1, \dots , N$).
- For each (ith) virtual population, the PK and/or PD endpoints are checked against the success criteria, specified as per Eq. 4 (${K}_{1},\dots , {K}_{M}$ and ${n}_{1},\dots ,{n}_{M}$), to compute:
  5 $$PoPS={M}^{^{\prime}}/M$$
  where M’ is the number of virtual populations that have met the success criteria.

### Implementation

**Fig. 1:**
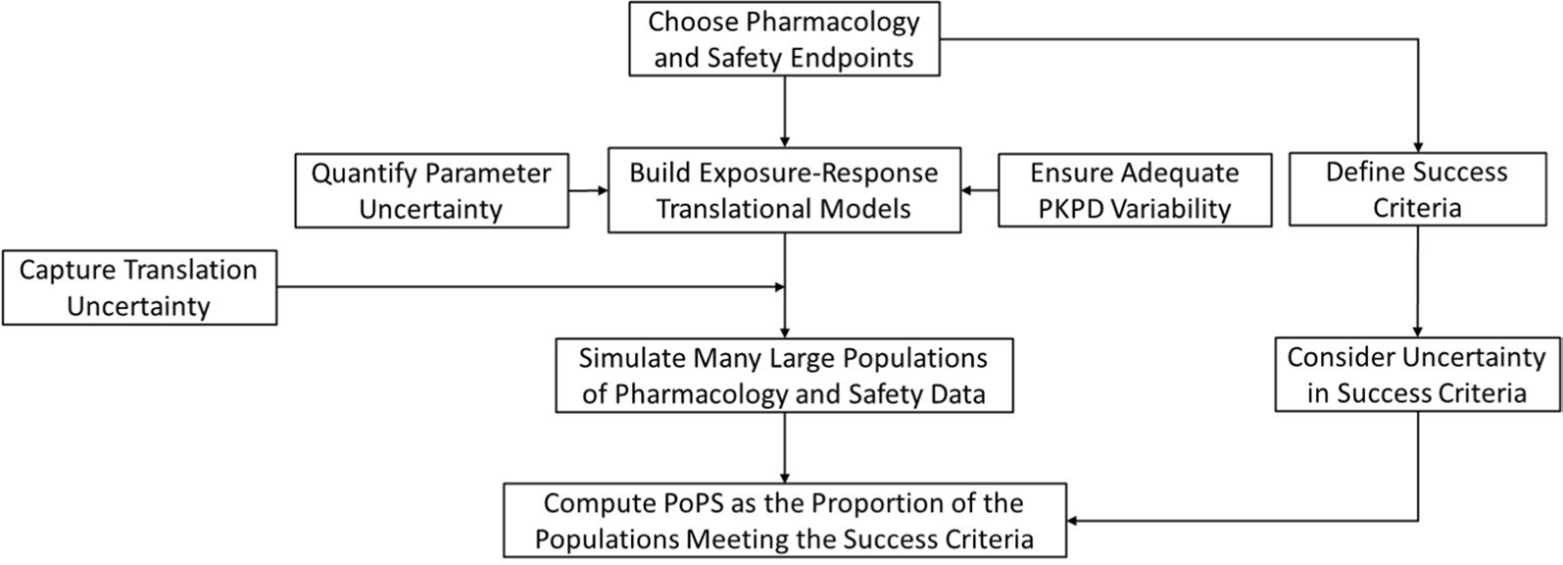
Guide for estimating PoPS (Probability of Pharmacological Success). Once the key pharmacology and safety endpoints are chosen, exposure–response models are developed with estimation uncertainty and between-subject variability for key parameters. Accounting for any translation uncertainty, the models are used to simulate virtual populations. Given the success criteria and their uncertainty, the simulated populations are used to compute PoPS

Figure 1 is a guide for PoPS estimation. Meaningful efficacy and acceptable safety are essential attributes for a successful medicine. For early-phase drug candidates without efficacy or safety data from patients, PoPS is informed by data on relevant pharmacology and toxicology endpoints. Based on these data, the endpoints’ success criteria and their associated uncertainty should be defined. The next step is to develop PKPD models for these endpoints, often based on data generated in vitro or in animals, capturing between-subject variability (BSV) and estimation uncertainty on key parameters. These models should be translated to patients, accounting for translation uncertainty. The translated models are then used to simulate populations of pharmacology and safety data, from which the PoPS is estimated based on the success criteria and their uncertainties.

### Selecting endpoints and defining success criteria

Deciding on whether a drug is good enough for further investment requires an a priori definition of ‘good’. For an early-stage asset, without data from patients, this is arguably the most critical, as well as the most challenging part of a credible PoPS estimation.

For the PoPS to be meaningful, the success criterion for the pharmacological endpoint—the magnitude of the required response and the proportion of the patients to achieve that magnitude—must be determined. Importantly, the required response should reflect the level that is expected to produce clinical efficacy; it should be defined based on scientific evidence, not intuition.

An administered compound produces efficacy typically by following the order: exposure—target engagement (TE)—signal transduction (ST)—pathway pharmacology (PP)—disease modulation (DM)—clinical efficacy. The endpoint for PoPS computation can be a PK one like in vivo maximum concentration (Cmax), relevant part of the area under the concentration–time curve (AUC), time to reach or time above a critical concentration, etc. It can also be a PD endpoint (TE, ST or PP) from in vitro, ex vivo or in vivo experiments. The area under the effect curve—integrating both PK and PD—has been proposed recently as an efficacy determinant, for anti-infectives and potentially other drugs [8].

Most of the drugs in our experience of PoPS assessment are of unprecedented mechanism; hence identifying an endpoint that can be successfully benchmarked for efficacy is usually the biggest challenge. Because the mechanisms of action of different compounds for same or similar indication often converge downstream of the above causality chain, choosing a distal endpoint (like PP or DM) instead of a proximal one (like exposure or TE) often provides opportunities to identify the required response level. In such cases, benchmarking is possible using existing data from another drug that has already shown evidence of efficacy. Ideally, a disease marker that is largely independent of drug mechanism and close to the efficacy endpoint of the drug-disease system should be used. The pharmacology success criteria of the case studies described below show that the benchmarking can be based on genetic evidence (Case 1), in vivo and human ex vivo experiments combined with medical and biological insights (Case 2), clinical data from drugs of a similar mechanism (Cases 3 and 4), or patient data in a similar indication (Case 3).

The toxicity mechanism is often less understood, consequently the success criterion for the safety aspect is most likely (though not always) empirically specified based on drug exposure limit in animals. The exposure limits could be (again, not always) those at NOEL (No-Observed-Effect Level), NOAEL (No-Observed-Adverse-Effect Level) or a low OAEL (Observed-Adverse-Effect Level) identified in animal studies. A safety margin relative to the exposure limit and the proportion of the population allowed to exceed such limit are generally decided depending on the animal species relevance, and on the seriousness, detectability, and reversibility of the dose-limiting toxicity. Where the dose-limiting toxicity is unintended/exaggerated pharmacology, a more mechanistic approach than using the exposure at NOAEL, e.g., defining the safe limit of the pharmacology, can be taken (Case 1).

The proportion of the patients who need to achieve the required pharmacology and/or staying within the safety limit is an important element of the PoPS specification. For pharmacology, if there’s strong theoretical or benchmarking evidence that the required level as a population average can produce clinically relevant efficacy in that population, it might be appropriate to require 50% patients to achieve this level. Otherwise, a higher proportion would be desired. For safety, factors to consider include the robustness of the safety limit, the nature of the toxicity and the benefit-risk ratio for the indication. For example, if the safety limit is the NOAEL, and the NOAEL level is far below the minimal OAEL, it might be safe to require 50% patients to stay within the NOAEL. Otherwise, a high proportion might be required.

Because the understanding in the pharmacology needs and safety limit is usually poor for early-phase compounds, especially for unproven mechanism, it is important to capture the uncertainty in these criteria via prior distributions and/or scenario evaluations while computing PoPS.

### Modelling and translating the exposure–response relationship

Based on the experimental data of the selected pharmacology and safety endpoints—internally generated or mined from literature—pharmacometric models of exposure–response relationships need to be developed. Structurally, these models should be sufficiently mechanistic to not violate the underlying understanding of the drug-disease system. Mathematically, they should be as simple and as transparent as possible, to aid discussion and reality check with the members of the project team. The uncertainty in PKPD estimation should be quantified (Fig. 1). This can be achieved generally by capturing the standard errors of the relevant model parameters and including their values in the subsequent PoPS simulations.

The exposure–response data generated in vitro, ex vivo from human tissues, or in vivo from animals need to be translated to humans in vivo. General principles of translational exposure–response modelling should be followed [9–12]. Translational uncertainty that can significantly impact the outcome of PoPS should be captured (Fig. 1). As illustrated in the case studies below, data from human tissue can be used to develop an in vivo model by dynamic modelling (Case 2); data from animals can be scaled allometrically to humans (Cases 2 and 3) and adjusted by tissue distribution of the drug (Case 3); potency or drug–﻿target binding affinity should be species-corrected (Case 3); and non-specific binding in circulation should be calibrated for PK and for PD, wherever appropriate (Cases 1, 2 and 4).

Because PoPS is defined as the probability that a proportion of patients can be effectively and safely treated, it is important to capture the BSVs in relevant PKPD model parameters (Fig. 1). When a variability is known to exist but its value unknown, an experience-based assumption can be made. For oral clearance, BSV is typically 25–30% for drugs of high solubility, high permeability and low hepatic clearance; whereas drugs with low absorption or high first pass often have higher, perhaps 50–80%, BSV. When translating in vitro exposure–response to in vivo, it is prudent to apply higher BSV to in vivo PD parameters than estimated from in vitro. It is not necessary to assign BSV to all parameters; judgement should be used to ensure reasonable overall variability. For example, when using a highly mechanistic model with a large number of parameters, assigning experimentally observed BSVs to most or all parameters would inevitably inflate the biological variability [13]. It is also appropriate to include covariance between clearance and volume parameters (both are positively related to body size), and potentially between baseline and maximum PD effects E0 and Emax (depending on the nature of the measurement and model parameterisation). The BSV in PK and PD parameters can also be higher in patients than in healthy people, due to a range of intrinsic and/or extrinsic factors. When a variability important to PoPS computation is highly uncertain, a distribution function for its value can be applied to reduce the risk of grossly over- or under-estimating the PoPS.

### Simulating virtual populations and computing PoPS

The success criteria usually include both the intervention needs and the safety limit. If the evidence for the intervention needs or safety limit is unclear, an uncertainty distribution should be used (Fig. 1). The nature of this uncertainty distribution should be decided jointly by the biologist, toxicologist, clinician, biomarker expert and translational pharmacologist. Importantly, the uncertainty in the success criteria, just like the success criteria themselves, should be based on scientific evidence supported by deep understanding of biology to minimise subjectivity. As examples, in Case 1, the literature-reported diverse range of the need for normalising the activity of the drug target in the brain was captured; in Case 2, the human relevance of the NOAEL values from three animal species were considered differentially, based on the understanding of the species difference in target expression; and in Case 3, a range of potential required pathway pharmacology was used, according to reports on compounds of related mechanisms and for similar indications. Credible PoPS computation relies on reasonable quantification of these uncertainties: over-estimation of these uncertainties is likely to under-estimate the PoPS, and vice versa.

For PoPS computation, the translational exposure–response model is used to simulate the dose–response data of pharmacology and toxicity endpoints in virtual patient populations (or large trials), incorporating PKPD estimation and translational uncertainties as well as BSVs. In essence, the uncertainties lead to difference between populations; and the BSVs lead to difference among patients in each population.

Each simulated large trial is then checked against the success criteria (considering the uncertainty in the criteria). The proportion of the trials meeting the success criteria at a given dose (or regimen) will be the PoPS at that dose (or regimen).

## Case studies

Four cases here, each for a different therapy area, showcase how the PoPS approach was applied at decision gates from preclinical candidate selection (Case 1) to FiH (Cases 2 and 3) and clinical proof-of-concept (PoC, Case 4). To protect proprietary information, the compounds involved have been anonymised. Nonetheless, we believe sufficient details are described to illustrate key aspects of situational application of the PoPS approach, from how the pharmacology endpoint is identified and how the success criteria are defined, to how uncertainties in the drug-disease system were considered and how PoPS was computed to capture these uncertainties.

### Case 1: select a preclinical candidate for neurodegeneration [7]

A full report of this case is published [7]. Two lead compounds (A and B) for a neurodegenerative disease were ready for preclinical candidate selection. The target is expressed both in the brain and peripheral tissues; the elevation of the drug target activity in the brain is genetically linked to the disease. While target inhibition is a potential treatment, in-class data from animals had shown pharmacology-driven lung and kidney toxicity at high levels (> 90%) of peripheral target inhibition; therefore, the intervention need was defined as normalizing the brain target activity but preserving enough activity in peripheral tissues to mitigate the pharmacology-related safety risk. A decision was required to choose one of the two compounds for further preclinical development. However, before PoPS estimation, the two compounds could not be clearly differentiated, given both compounds demonstrated enough potency to inhibit the target activity to the normal level in the in vivo animal studies. Because the therapeutic success relied on balancing target inhibition in the brain versus in the periphery, the target activity, as measured by a blood biomarker, was the endpoint for both pharmacology and safety aspects of PoPS. The PoPS was specified as the probability for ≥ 80% of patients to achieve the desired balance while putting < 5% of patients at risk of over-inhibition.

One-compartment PK model in human, with first-order absorption and elimination was constructed for each compound, where key parameters including apparent oral clearance (CL/F) were predicted from multiple animal species. A PK/PD model for central and peripheral inhibition was further developed for each compound. The central and peripheral inhibitions were described using simple Emax models revealed by animal experiments, where the drug concentration leading to 50% of Emax, EC_50_, was substituted by estimates from in vitro human cell lines, the Emax was assumed to be 100% target inhibition, and the steady-state average free concentration considering brain-plasma unbound drug portion (*k*_p,uu_) was applied for the model. Translational uncertainty in *k*_p,uu_ was addressed by applying a uniform distribution to the values estimated from multiple species: 0.45–0.75 for compound A and 0.35–0.50 for compound B. The key PK and PD parameters—CL/F, *k*_p,uu,_ and EC_50_ were assumed to follow a log-normal BSV distribution, per established practice. Both compounds are Biopharmaceutical Classification System (BCS) class II compounds with limited solubility and high permeability; hence a moderate BSV (CV% = 30%) was assigned for CL/F, given 20–40% CV% usually estimated for this class [14–16]. The EC_50_ was assigned a 30% BSV, as estimated in vitro.

**Fig. 2:**
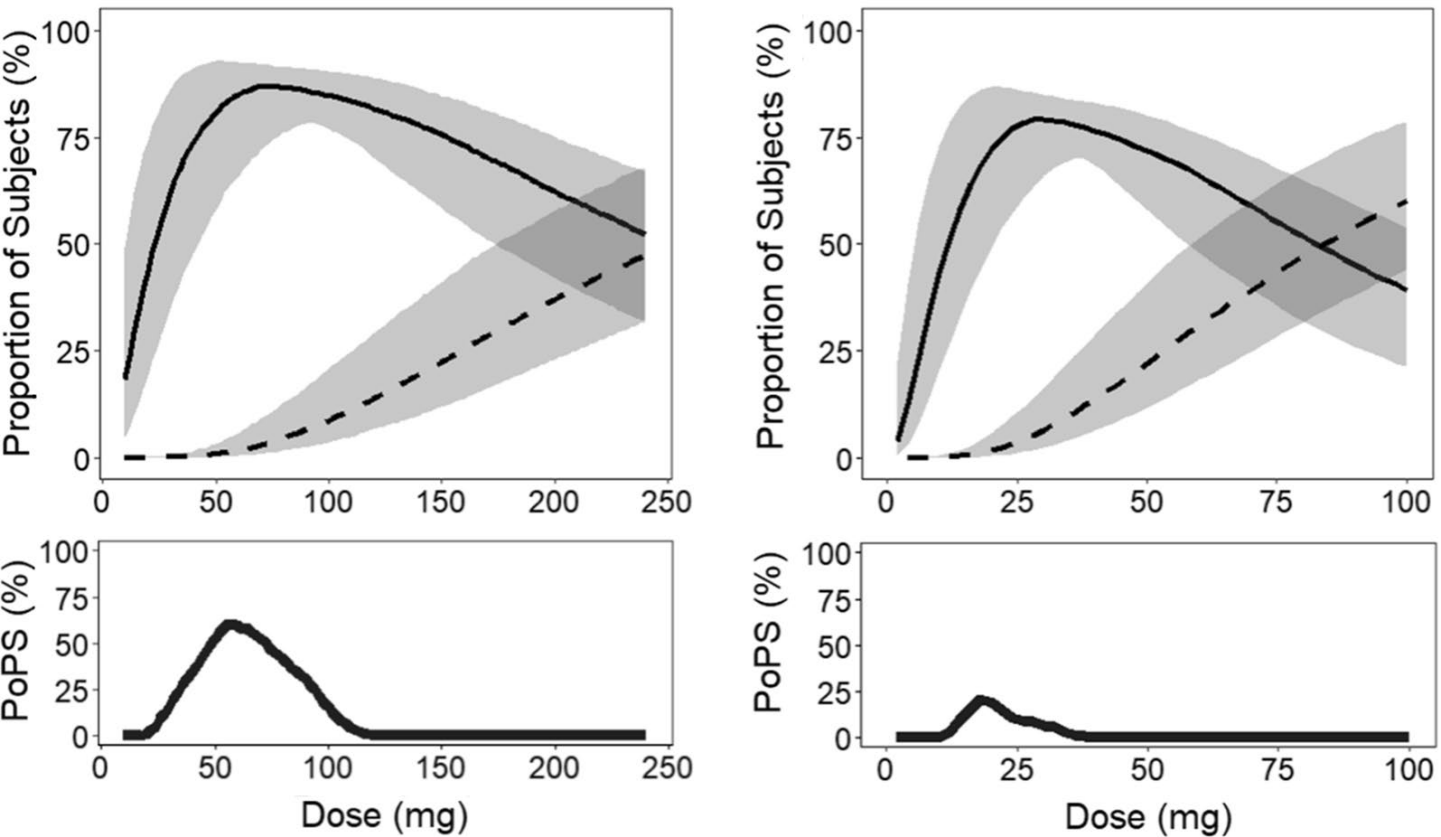
Case 1. Probability of pharmacological success (PoPS) for two compounds against neurodegeneration—Compound A (left) and Compound B (right). Upper panels: proportion of patients with sufficient central inhibition and peripheral preservation (solid curves), and proportion of patients with insufficient peripheral preservation (dashed curves); the lines are the medians of simulations; the bands reflect the 90% predictive intervals of simulations. Lower panels: PoPS of the two compounds

Clinical data were simulated for 1000 trials of 1000 subjects each using this model, including the BSVs and the relevant translational uncertainties described above. For PoPS computation, the uncertainty in the intervention needs—to normalise the target activity—was considered using the literature-reported range of the target elevation (1.5–3.0 folds) in the disease; whereas the range of *k*_p,uu_ values determined from multiple species was the translation uncertainty. A unique combination of the two parameters was assigned to each simulated trial. In each of the 1000 simulations, the patients with desired benefit-risk profile were the ones with sufficient central inhibition (normalized target activity) and peripheral preservation (preserving ≥ 10% activity), and the patients with safety risk were the ones with insufficient peripheral preservation (Fig. 2 upper panels).

The results of this assessment showed the maximum proportions of patients achieving the required brain target activity inhibition and peripheral preservation were similar for the two compounds- 87% vs 79% (Fig. 2 upper panels), which still could not suggest a clear differentiation. However, when both efficacy (≥ 80% of patients with desired benefit-risk ratio) and safety criteria (< 5% of patients with safety risk) were considered together, the maximal PoPS was 61% for Compound A (at 56 mg daily dose, Fig. 2 lower left panel), and 21% for Compound B (at 18 daily dose, Fig. 2 lower right panel). Given the clearly higher PoPS of compound A, it was selected over compound B for further development.

This case study provided an example that PoPS led to an unambiguous decision at the stage of preclinical candidate selection, despite the fragmented nature of the drug-disease system knowledge and multiple uncertainties. The PoPS findings also provided insight for future compound discovery and guidance on clinical trial population selection; these were discussed in the original paper [7].

### Case 2: commit to FiH of a molecule for a heart condition [17]

A compound for a heart condition was approaching FiH decision gate. Based on preclinical evidence, the molecule is expected to produce efficacy by inducing the production of a protein. The concentration–response of the protein’s mRNA was assessed in human blood ex vivo, while the exposure–response of the efficacy endpoint was evaluated in a mouse model. Toxicokinetic experiments were performed in three animal species, where PK and NOAEL were established. The PoPS was specified as the probability that ≥ 90% of patients achieve adequate pharmacology, and ≤ 5% exceed the safety limit.

The protein mRNA, a biomarker reflecting signal transduction, was selected as the pharmacology endpoint, because it can be measured in healthy volunteers to inform dose selection in patients. A transduction PKPD model [18] was used to translate ex vivo data into an in vivo relationship by combining it with the predicted human PK (allometrically scaled from preclinical data).

To benchmark required pharmacology, an Emax relationship between PK and the efficacy endpoint was identified based on the mouse efficacy model and was scaled to human accounting for plasma partitioning and protein binding differences. The pharmacology and efficacy were bridged by PK: the required pharmacology was defined based on the exposure which was in turn corresponding to relevant efficacy.

Estimation uncertainty was included on key parameters, i.e., protein mRNA Emax and EC_50_. The percentage relative standard error (% RSE) of estimate derived ex vivo was used to define prior width; furthermore, estimation correlation between Emax and EC_50_ was incorporated into prior definition. Translational uncertainty was also included on PD BSV, by assuming three equally probable scenarios of low, medium, and high protein mRNA BSV.

Moderate PK BSV (CV% = 30%) was included on key parameters (central clearance, central volume of distribution, absorption rate constant). The BSVs in baseline (E0) and maximum increase in protein mRNA (Emax), both estimated ex vivo, were included.

The pharmacology need was defined as approximately 2–10 folds protein mRNA increase, based on medical and biological evidence that this level of pharmacology could produce meaningful efficacy. The safety limit was based on the molecule’s exposure being below the NOAEL from the three preclinical species.

Uncertainty was included on pharmacology need and safety limit. Data from animal models show that a 2–fourfold increase in protein mRNA was usually required to achieve meaningful efficacy, while higher values were only occasionally needed. Therefore, a beta distribution with mode at 2 fold (with decreasing probability towards values > 10 fold) was used to describe the required pharmacology. To reflect the knowledge at the time about the relevance of the three preclinical species (rat, dog, monkey), a weighted prior (60:30:10) was applied to the safety limit.

**Fig. 3:**
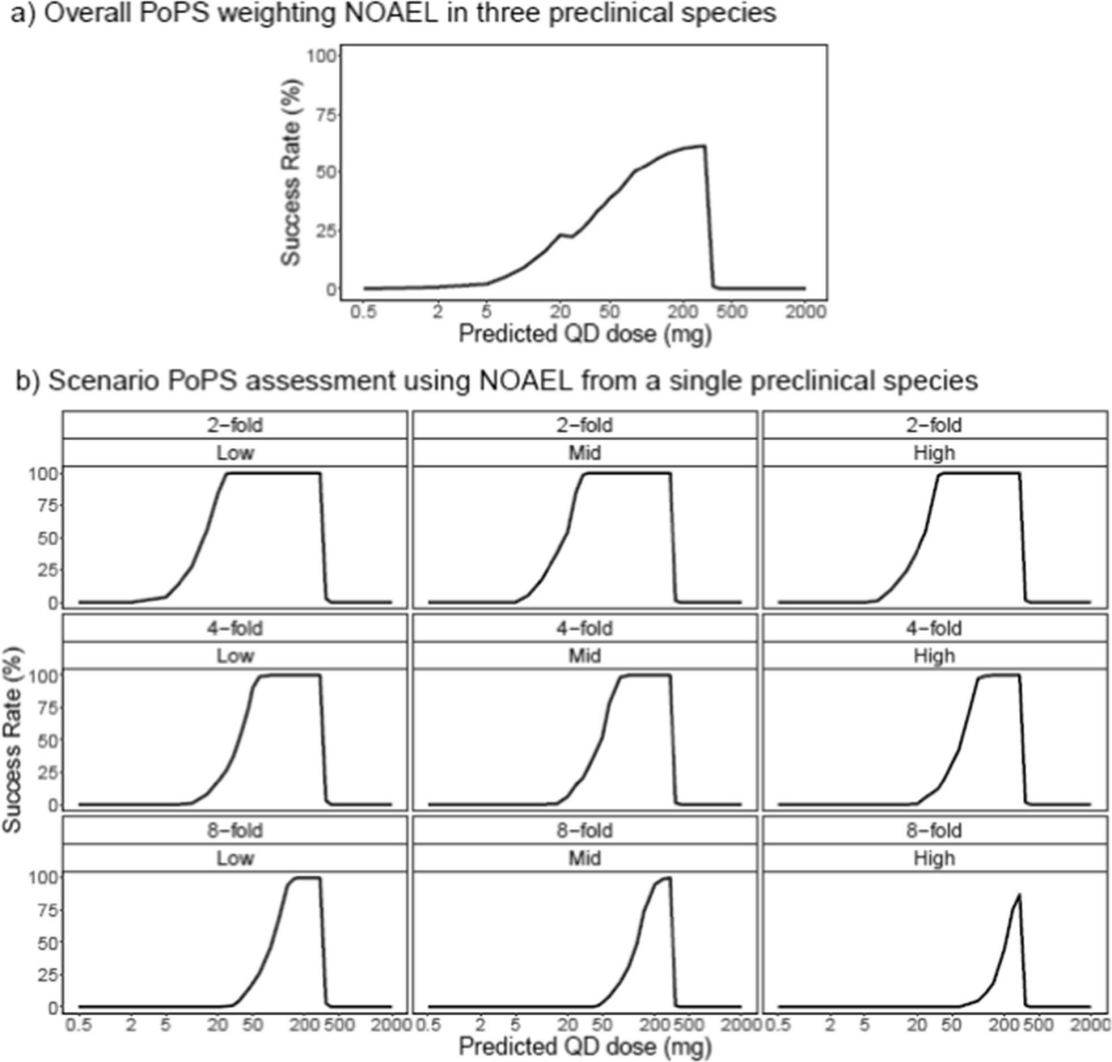
Case 2. **a** Initial overall PoPS weighting NOAEL in three preclinical species; **b** Scenario assessment of final PoPS per biomarker fold-increase (2-, 4-, or 8-fold) and variability level (low, mid, or high) using NOAEL from the single most relevant preclinical species

The PoPS was computed by simulating the mRNA level (for pharmacology) and plasma exposure (for safety) for 1000 virtual populations, each with 1000 patients per dose level, initially assuming priors outlined above. Subsequent toxicology assessments and expert consultations concluded that dose-limiting safety findings in rat and dog were unlikely to translate to human at the proposed clinical dose range. Therefore, the PoPS was re-estimated using the safety limit from monkey only. Scenario analysis was conducted, considering varying pharmacology requirement (2- to 8-fold biomarker increase) and protein mRNA variability level (low to high) (Fig. 3).

The first PoPS assessment provided a peak ~ 60%, at 300 mg QD dose (Fig. 3a). In the subsequent scenario analysis, the updated PoPS peaked at ≥ 90% (Fig. 3b).

In this case study, PoPS effectively integrated multi-source data, weighting their relevance through close interaction with experts, and outlined key assumptions and uncertainty sources. It provided an early understanding of the therapeutic potential of the molecule thus enhancing the confidence and clarity of the commit-to-FiH. Once clinical PK and protein mRNA data from the FiH are available, the PoPS will be updated to inform the decision to progress to subsequent patient trials.

### Case 3: commit to FiH of a molecule for an auto-immune disease [19]

The molecule is a humanised IgG1 monoclonal antibody, for an autoimmune disease, approaching FiH decision gate. The binding of the drug to a soluble cytokine (TE) prevents the cytokine’s interaction with its receptors located on an autoimmune T-cell subset, reducing the expression of an intracellular protein (i.e., PP), and consequently decreasing the count of those autoimmune T-cells. The PoPS for the asset was specified as “the probability that > 50% of the subjects achieve required protein reduction in the target tissue, while > 50% have systemic drug exposure within the relevant safety limit”.

Modelling was performed using serum data of drug exposure, TE, and PP in monkeys. A target mediated drug disposition (TMDD) model was used to describe the TE. The PP was characterized by an indirect response model, where protein turnover in response to serum drug concentrations was characterized by an Imax model. The TE and PP models were translated to humans via allometric scaling of PK parameters and cross-species adjustment of baseline target level. The parameters describing protein turnover in monkeys were considered similar in humans. For PoPS computation, the PP endpoint was chosen over the TE endpoint because, being a downstream measure, it enabled the identification of the required pharmacology based on literature data of two compounds (one with a similar mechanism and for a similar indication; the other with a different mechanism though for the same indication). The PP model was extrapolated to target tissue by adjusting for drug’s distribution to the tissue.

For the PoPS, it was crucial to include uncertainties at three levels i.e., estimation of PKPD parameters, translation of pharmacology from blood to tissue, and therapeutic need. Estimation uncertainties (% RSEs) were incorporated on PD parameters for drug’s efficacy (Imax) and potency (IC_50_) as well as the rate of protein degradation. Correlations among PK (clearances and volumes of distribution) and PD (Imax and IC_50_) parameters were included using the estimates from respective covariance matrices. The PP in the tissue was used as a metric for pharmacological effect. Because the drug effect occurs predominantly in the tissue but is measured in the blood, a blood-to-tissue translational uncertainty was included based on the estimated plasma-to-tissue distribution coefficient. The uncertainty in therapeutic need was incorporated via a uniform distribution (70–95% inhibition of the cytokine-driven protein), informed by the two benchmark drugs. The safety limit was derived from exposure at the NOAEL as observed in monkeys.

**Fig. 4:**
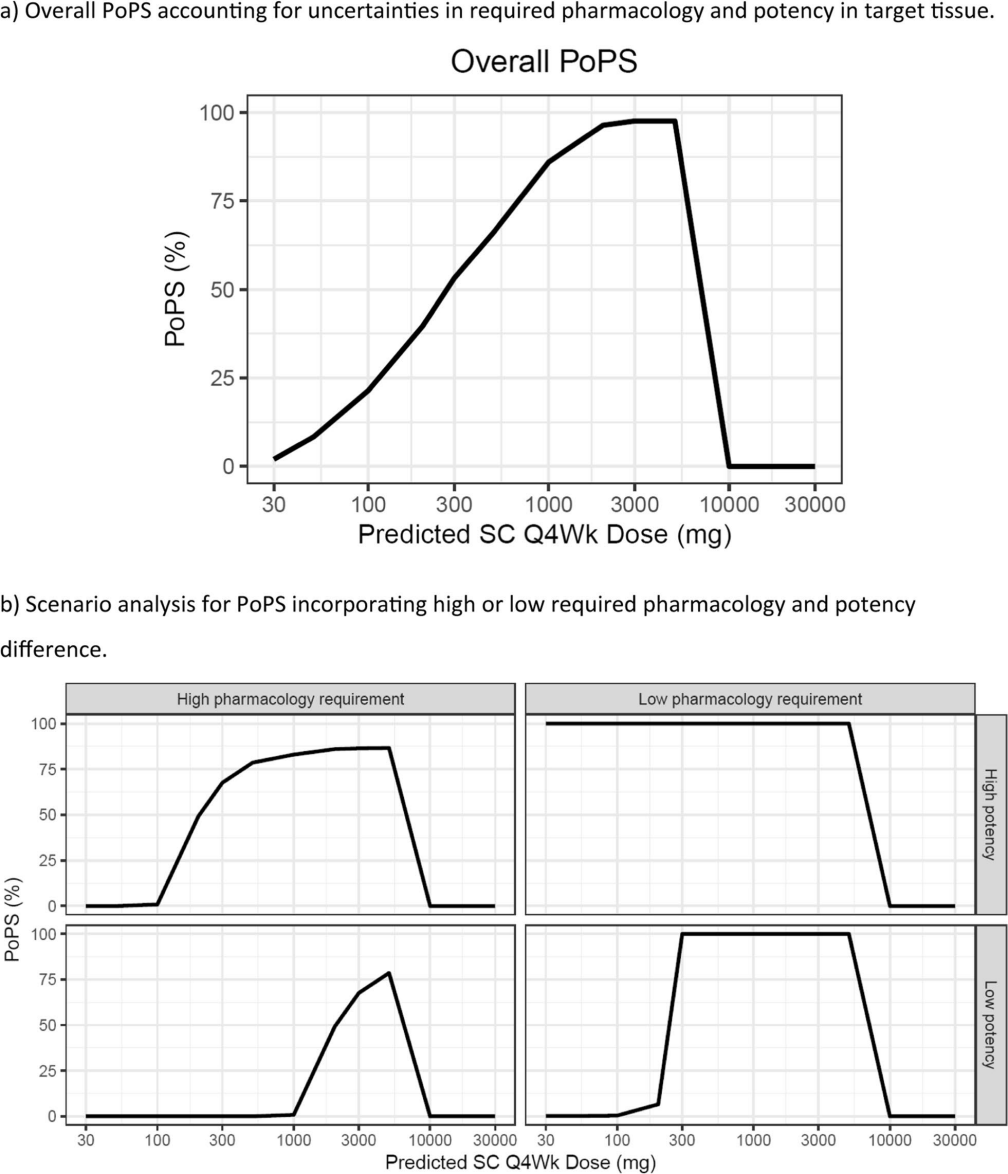
Case 3. Overall PoPS and Scenario analysis for commit to first-in-human of a molecule for an auto-immune disease

Clinical PP simulations were conducted for every-four-week (Q4W) dosing, including BSVs on PK and PD parameters as estimated from monkey data. In total, 500 trials (accounting for PKPD estimation and translational uncertainties) were simulated, each trial having 500 individuals (accounting for BSV). Using prior distributions for the pharmacology requirement, the overall PoPS was > 95% (Fig. 4a). Scenario analysis was also conducted (Fig. 4b). Under the most relaxed conditions, i.e., high potency in tissue and low requirement for pharmacology, PoPS was > 95%. Under the most stringent conditions, i.e., low potency in tissue and high requirement for pharmacology, a moderate PoPS of ~ 75% was estimated. Both for overall PoPS and scenario analysis, at doses > 5000 mg Q4W, a sharp decline in PoPS was expected, due to the exposure exceeding the safety limit.

Choosing the PP as the pharmacology endpoint for PoPS computation was a cornerstone for this case. It allowed benchmarking of required pharmacology using clinical data of drugs with a similar mechanism or for a similar indication (see above). The successful benchmarking in turn significantly expedited the program. The PoPS results established a high confidence in the drug’s ability to deliver required pharmacology within the desired safety limit, in this case leading to a firm decision of progression to FiH ahead of schedule.

### Case 4: commit to clinical PoC of an oral anti-bacterial [20]

A candidate antibiotic was at the decision point for committing resource to clinical PoC, following FiH where human PK, safety and tolerability were assessed. The efficacy endpoint for the PoC would be the standard measure of bacteria decline in the relevant biological matrix. The method of PoPS computation was different from the other cases described above and followed less closely the process outlined in Fig. 1. It was inspired by the probability of target attainment (PTA) approach, established in antibiotic research [21–23]. The PTA approach maps out the cumulative distribution of an efficacy-driving PKPD index in a patient population. The usual index is AUC:MIC ratio, Cmax:MIC ratio or Time > MIC (the proportion of the dosing interval when plasma concentration of the drug is above MIC), where MIC is the minimum inhibitory concentration for pathogen growth. Achieving a critical value of the cumulative distribution observed for an effective treatment is then considered as the requirement for treatment success.

To choose the appropriate pharmacology endpoint for PoPS and establish the required response level, we identified a drug from the same class that had demonstrated varying degrees of efficacy in multiple studies. A population PK model was constructed for the benchmark drug and used to simulate PK profiles, sampling over all estimated BSVs in these positive studies (N = 3000 for each study). The MIC distribution found in literature was fitted by a logistic function, which was used to simulate a large population (N = 3000) of values to be paired randomly with the simulated PK. Meta-analysis including the multiple studies showed that the strongest efficacy driver was AUC:MIC [﻿^20^], which was chosen as the endpoint for computing PoPS for the candidate drug. The distribution of the AUC:MIC for this benchmark drug at a target efficacy level served as the response level required for efficacy for the drug class.

To estimate the PoPS, a population PK model was constructed for the candidate drug using the FiH data. Variability in MICs was described by a log-normal distribution, fitted to proprietary data. The PK model was used to simulate a large population (N = 3000) of PK profiles for the highest proposed PoC dose, sampling over all estimated BSVs. MICs were subsequently sampled from the fitted log-normal distribution giving distribution of randomly paired AUC:MIC values (N = 3000). The distribution of AUC:MIC values for the drug was then compared to that for the reference drug at the (latter’s) target efficacy level.

**Fig. 5:**
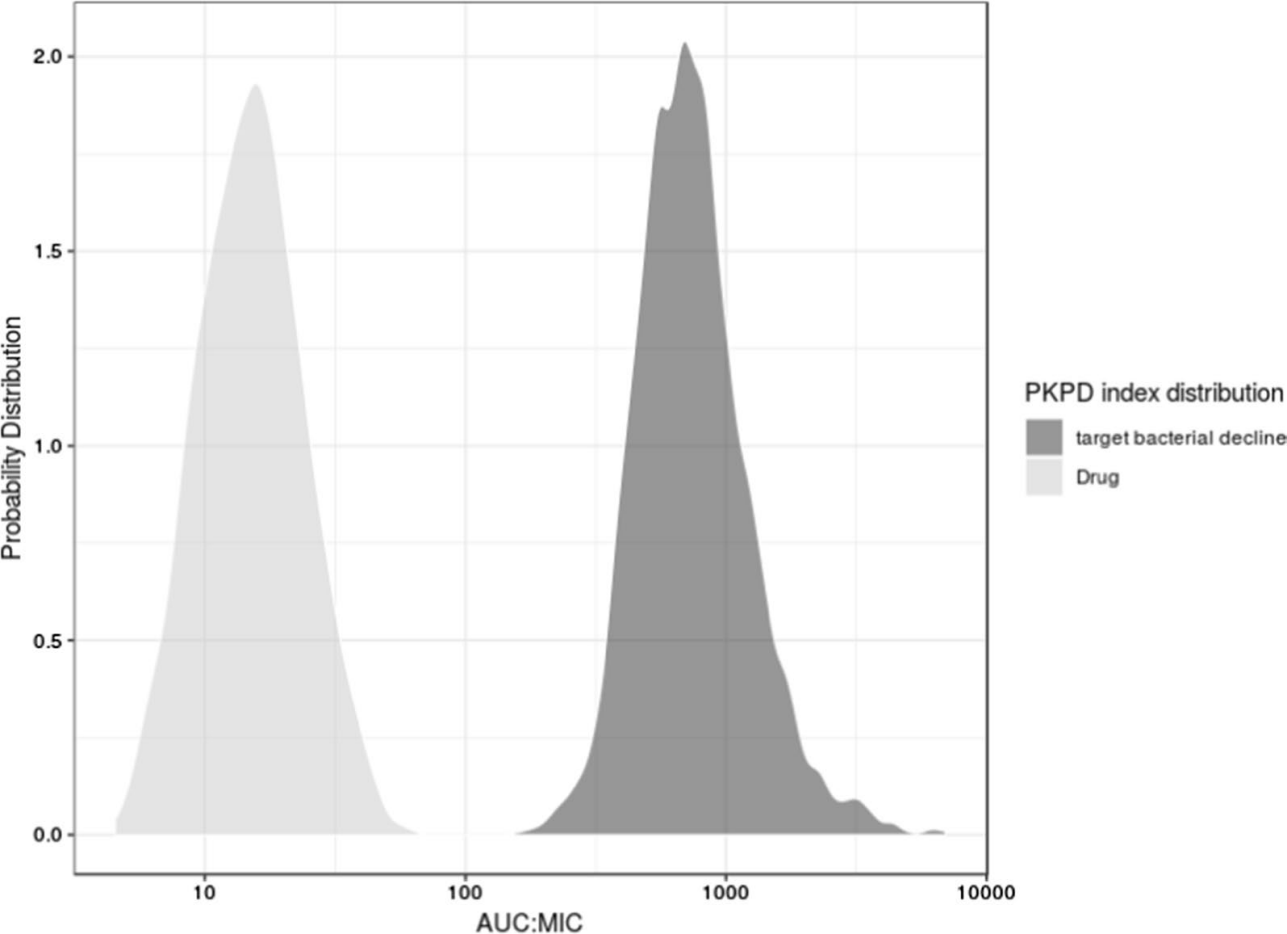
Case 4. Probability distributions of AUC:MIC for the candidate drug (left) and the benchmark drug regimen (right) that gave target mean bacterial decline. The lack of any overlap between the two distributions indicated negligible PoPS for the candidate drug

Uncertainties were not explicitly included in this PoPS calculation for multiple reasons. Under time pressure, we were pragmatic to not include PKPD uncertainties: uncertainty (%RSE) for PK parameters was well below 20% for both candidate and benchmark drugs, as both models were built from frequently sampled rich data; and uncertainty in MICs was considered to be less than two-fold, inherently due to the serial-dilution experimental design for their estimation. Furthermore, uncertainty in translation was not relevant as we used clinical data of an in-class benchmark compound for the same infection. Finally, uncertainty in pharmacology requirement (AUC:MIC) was not needed when the PoPS at the highest proposed dose turned out to be negligible when benchmarked for the target efficacy (Fig. 5).

This was a new framework for predicting the probability of achieving target pharmacological effect levels, applicable to any anti-bacterial drug where in-class benchmark is available. Because the PoPS—the probability of the drug reaching benchmark AUC:MIC values corresponding to the target efficacy—was found to be negligible even at the highest proposed dose, the onward internal funding for the molecule was deprioritised.

## Discussion

The importance of understanding the probability of success at trial level and for later-stage programs has been stressed [24] and several methodologies have been proposed [25–27]. Decisions whether to progress an early-stage asset remain particularly challenging, due to the complexity of multiple uncertainties in the drug-disease system. The computation of PoPS is a mechanism to identify, quantify, document, and integrate these uncertainties in a single term of probability to inform a resourcing decision, considering the needs of both pharmacological intervention and safety.

The above four examples at different early decision gates and across different disease areas (central nervous, cardiovascular, autoimmune, and anti-infective) show how PoPS situationally informed the decision. It effectively led to successful compound selection (Case 1), supported confident decision to progress (Case 2), accelerated development program (Case 3), and changed resourcing strategy (Case 4).

### An integral part of translational research

The process of estimating PoPS enables more meaningful non-clinical-to-clinical translation. The evaluation of PoPS as a function of dose means more rigorous clinical dose prediction. The examples demonstrate that credible computation of PoPS demands deep understanding of the drug-disease system, hence the PoPS framework has a role as an integral part of a program’s translation strategy.

The framework itself requires a pharmacology endpoint with quantifiable relevance to clinical efficacy. This forces deep understanding of the system—on disease pathology, animal model relevance, biomarker assay, drug mechanism and pathway, and even mechanism and pathway of drugs with similar mechanisms or for similar indications—which in turn enriches the knowledge of how well the drug will work. Meaningful computation of PoPS relies on relevant and high-quality data. Major data gaps or data of poor quality would diminish the value of the exercise. Via PoPS approach, key uncertainties in the system are identified and documented for transparency, exposing translational gaps and guiding the conception and design of future experiments. In this context, the scenario analysis (Cases 1, 2 and 3) is a useful tool for assessing the relative importance of each uncertainty and for identifying the need for further experiments to bridge the knowledge gaps.

A detailed analysis of Case 1 illustrated further versatility of this tool; much of this was the outcome of scenario assessments [7]. The PoPS exercise revealed insight on key characteristics required for future compounds—a valuable piece of back-translation to steer discovery efforts. It also identified the patient population that is likely to be most susceptible to the intervention—a clear demonstration of the power of the drug-disease framework towards precision medicine. Its dose–response function was useful for understanding the viable dose range, in connection with formulation development, cost-of-goods, pre-systemic toxicity or impurity discussion.

### A decision enhancer

Decisions must be made at risk whether to progress a compound. Traditionally, these decisions are made intuitively, when the strengths and weaknesses counteract against each other, and with further complications of knowledge gaps. Depending on an individual’s selective awareness, preference and bias, these decisions can be highly subjective and arbitrary.

A recent analysis of decision bias in pharmaceutical R&D suggested turning the implicit nature of decision making into an explicit one as a mechanism for improving decision quality [28]. The PoPS serves as a mechanism to integrate a compound’s relevant strengths and weaknesses, to identify the knowledge gaps, and to put reasonable levels of uncertainties to necessary variables. The uncertainty assessment is inevitably imperfect; however, the examples show that the process is evidence-based. This, compared to opinion-based alternative approaches, enhances the quality of the decision by increasing transparency and objectivity.

In our Research & Development organisation at GSK, the value of the PoPS approach in evidence-based decision-making is well recognised; its planning and evaluation are now expected for governance review of all assets at early-phase decision gates. A tool is being developed to facilitate its broader and more efficient implementation across projects [29]. In time, it would be interesting to assess PoPS’ predictivity—what proportion of early-stage drug candidates with a high PoPS eventually show favourable benefit-risk ratio in late-stage clinical trials.

In a way, the PoPS process defines what a promising compound should look like (pharmacologically) and estimates how good the compound being assessed is. But how good is good enough, hence worthy continued investment of resource, is a business decision. Factors to consider include the level of unmet medical needs, the competitive landscape and the organization’s tolerance of investment risk.

### PoPS evolves as the asset journeys through

Drug development is a journey of knowledge building; the PoPS for an asset is expected to progress as the knowledge grows. Case 2 is a good example: when emerging evidence suggested some animal species were not relevant, the uncertainty in safety limit was reduced and PoPS re-calculated accordingly. As a compound moves from CS to FiH to PoC, the data source of its pharmacology and safety typically evolves from animals to healthy people to patients. The relevance of the endpoint to disease tends to increase from in vitro to in vivo and from shorter to longer terms. For example, at the decision gate for progressing to FiH (Phase 1a), the pharmacology data are often generated in vitro or from animals. Such data need to be translated to human before the success criteria can be applied. When the asset approaches Phase 1b (PKPD in patients) or Phase 2a (clinical PoC), PKPD data are usually available from healthy people in Phase 1a. Such data would be adapted to patients considering disease conditions and applied to the success criteria relevant to the PoC decision. Of note, the endpoint itself used for PoPS may well be different from one decision gate to the next. The pharmacology endpoint may evolve from pathway pharmacology to a disease biomarker; and the safety endpoint may evolve from the NOAEL observed in animals to emerging adverse event in the FiH. As such, the translation uncertainty can be expected to reduce as well. In essence, the PoPS is a decision tool based on the information available at the time; both the choice of the endpoint and its success criteria could be revised as appropriate.

### A cross-discipline exercise

Each of the cited cases reflected close collaboration among the involved translational pharmacologist, biologist, physician, toxicologist, biomarker specialist, project leader and project manager. Such collaboration was essential, from formulating biologically meaningful and clinically relevant translational strategy, to delivering sound and credible PoPS estimation on time for the progression decision.

Domain knowledge is essential: identifying the appropriate pharmacological endpoint, understanding its relevance to clinical efficacy, and defining the required intervention level demand deep understanding of disease biology and drug mechanism. Building translational models and conducting stochastic simulation to compute PoPS involve pharmacometric skills, including the ability to reality-check the biological meaning of the model structure and the implication of the uncertainty and variability assignments.

Early planning is key, to set realistic expectation of the time, budget and human resource needs, to allow time for developing relevant PK and PD assays, to design and conduct dose–response experiments, to ensure rigorous model construction and consultation, and to allow for robust expert discussion and broader team alignment on the results before investment board review.

Although PoPS is aimed to assess the asset-level therapeutic potential, the approach can also be applied to inform the probability that a clinical trial can achieve its success criteria defined in terms of a PD or (surrogate) efficacy endpoint. In this context, the assumption around the uncertainty in the drug-disease model may serve as a Bayesian prior to inform trial design, treatment regimen or sample size; relevant patient covariates in the model can inform inclusion criteria, enrichment strategy or treatment allocation. Maximising the benefit of such trial-level implementation of the PoPS approach demands shared ownership among quantitative (translational pharmacology, pharmacometrics and statistics), clinical, operational, and regulatory disciplines.

## Conclusion

The PoPS approach aims to holistically quantify the overall potential of a molecule for its intended indication, on balance of pharmacology and safety. It is a powerful way to deepen the translational understanding of a drug-disease system. It serves to integrate multi-dimensional data and identify knowledge gaps. Its ability to capture multiple assumptions explicitly, quantitatively, and transparently in a single probability term makes it a promising tool to inform evidence-based rational progression decisions for early-stage drug candidates.

## Data Availability

Not applicable. No data from humans or animals are reported.
